# A Suspected Vestibular Schwannoma with Uncharacteristic Growth Dynamic and Symptom Severity: A Case Report

**DOI:** 10.7759/cureus.2024

**Published:** 2018-01-05

**Authors:** Felix Ehret, Alexander Muacevic

**Affiliations:** 1 Radiation Oncology, European CyberKnife Center Munich; 2 Radiosurgery, European CyberKnife Center Munich

**Keywords:** vestibular schwannoma, cyberknife, tumor growth, neurological symptoms, metastasis, radiation surgery, radiation oncology, neuro-radiology

## Abstract

Vestibular schwannomas are mostly sporadic, neuroectodermal, benign tumors of the myelin-forming cells of the vestibulocochlear nerve. Typical initial symptoms of vestibular schwannomas often include unilateral hearing loss, tinnitus, vertigo, and headaches. As schwannomas characteristically show a slow growth rate and various symptoms, different therapeutic approaches are possible, ranging from a watchful waiting strategy to radiation therapy and neurosurgical tumor removal. In addition, these treatment options should be evaluated carefully and assigned individually to the patients’ needs and symptoms while respecting the feasibility and possible outcome of the chosen treatment. We present a 69-year-old patient with an atypical, severe symptom constellation and tumor growth dynamic. The planned treatment of the schwannoma with radiosurgery revealed an unforeseen change of focus. Most notably, this case emphasizes the importance of a thorough radiological and patient-orientated assessment.

## Introduction

Vestibular schwannomas are the most common extra-axial tumors of the posterior fossa in adults. They represent approximately 85% of the tumors located in the cerebellopontine angle [1]. The incidence of the vestibular schwannoma is approximately one per 100,000, displaying a peak in the group of the 65- to 74-year-olds with nearly three per 100,000 [2]. Approximately 3300 new vestibular schwannomas are diagnosed each year in the United States [2]. As the sheer appearance of a schwannoma is no indication for treatment, various options in the management of the tumor are possible. Typically, three approaches are available and include a watchful waiting strategy (“wait-and-scan”), radiation therapy, and neurosurgical removal of the tumor. In regard to the general condition of the patient, symptoms, tumor size, and growth rate, the best suitable approach should be chosen. Due to the typical radiological appearance and morphology of schwannomas, a magnetic resonance imaging (MRI) scan is the gold standard for observation and can usually confirm the suspected diagnosis as well as possible progress. In addition, schwannomas represent a large group of incidental findings during MRI scans [3-4]. Unilateral hearing loss and tinnitus majorly portray the clinical picture of a newly diagnosed schwannoma. Further common symptoms include mild to moderate vertigo, headaches, and light-headedness [5]. Moreover, neurologic symptoms can occur, which range from facial sensitivity disturbances and visual blurring to a palsy of cranial nerves. However, these neurologic symptoms are linked to the tumor size and typically associated with bigger tumors [5]. In terms of the growth of vestibular schwannomas, the average growth rate is approximately one millimeter (mm) per year. Schwannomas, which are shown to grow, have an average growth of three mm per year [6]. We present a case of a rapidly growing tumor of the internal auditory canal with severe symptoms, including a grade IV facial palsy (House–Brackmann score).

## Case presentation

We describe a case of a 69-year-old man in good general condition (Karnofsky Index 90% / World Health Organization (WHO) / Eastern Cooperative Oncology Group (ECOG) performance status 0) with worsening hearing ability in the right ear and vertigo over the past nine months. Relevant pre-existing conditions in his medical history include a chronic obstructive pulmonary disease (COPD), gastritis, type 2 diabetes, arterial hypertension, and atherosclerosis of the left anterior descending coronary artery (LAD), a myocardial infarction 14 years ago and a recent symptomatic re-stenosis of the LAD. Furthermore, the patient suffered from a low-grade prostate cancer (TNM Classification of Malignant Tumors (TNM) cT1 cN0 cM0, Grading (G) 1, Gleason 2), which was initially diagnosed and treated in the year 2006. He underwent radiation therapy in the form of interstitial low dose rate brachytherapy (target volume 145 gray (Gy), 846.6 megabecquerel (MBq)). Currently, there is no evidence of a recurrence of the tumor. Due to his impaired hearing in the right ear, the patient underwent hearing screening. The results of the test showed a reduced hearing ability in the right ear, in a frequency range from 0.125 to 8 kilohertz. The median loss of hearing was 42 decibels (dB) on the right side in contrast to the left side with 26 dB. The supervising physician suggested a radiological presentation to further explore his condition.

**Figure 1:**
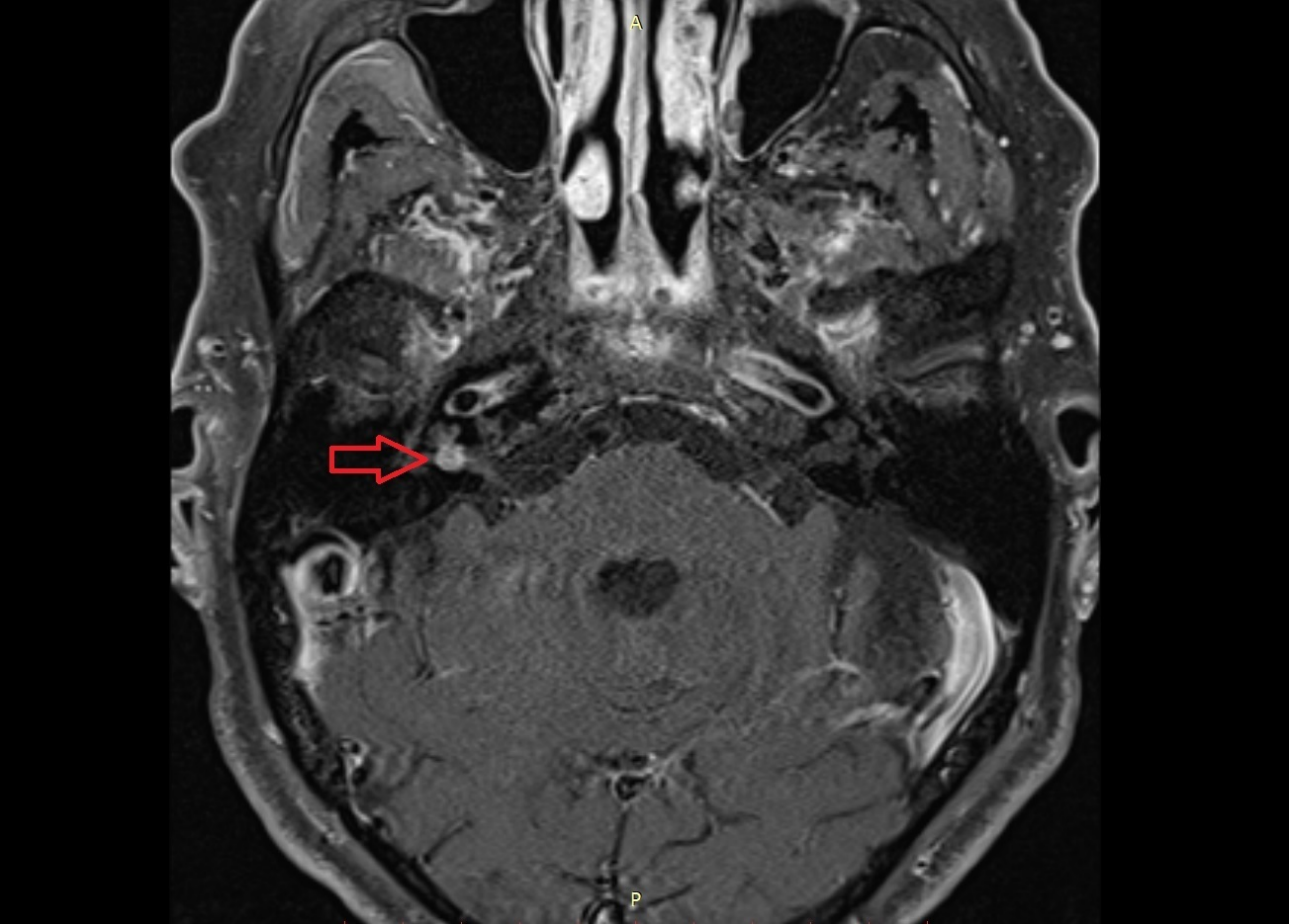
MRI scan. The image shows a round, solitary tumor with a size of 5 x 5 mm inside the right internal acoustic meatus. MRI: magnetic resonance imaging

The patient then underwent an MRI scan, which revealed a typical contrast-enhancing structure with a size of 5 x 5 x 4 mm in direct proximity of the right 7^th^ and 8^th^ cranial nerves, deep inside the right internal auditory canal. The radiologist classified the structure as a vestibular schwannoma, which could have caused the hearing loss and vertigo (Figure 1) [5]. In addition, no other suspect lesions were found and described.

To clarify further treatment options, the images and medical history were sent to the European CyberKnife Center in Munich, Großhadern. Due to the moderate hearing loss and the small tumor, the supervising physician recommended a watchful waiting strategy, including an MRI scan and hearing test follow-up with a re-evaluation in approximately six months. However, he classified the tumor as treatable with the CyberKnife (Accuray, Sunnyvale, California), a high-precision radiosurgery system, using a small, six MV linear particle accelerator. It is commonly used for the treatment of small benign and malign tumors and represents an excellent tool to treat vestibular schwannomas [7].

Two weeks after the evaluation of the patient’s symptoms and medical documents, the patient presented himself personally at the European CyberKnife Center for counseling.

Twelve days after the patient presented himself at the CyberKnife Center, he suffered from aggravating headaches, despite his medication of gabapentin, metamizole, codeine, and paracetamol, which was prescribed by a neurologist since the symptom onset several days ago. Moreover, he had severe pain feelings located in the left buttock, which spread to the ipsilateral foot. Additionally, he showed a high-grade facial nerve palsy on the right side and requested the urgent need for treatment.

To re-evaluate the situation and to find the possible morphologic equivalent for the short-term worsening of the patient, he underwent another MRI scan.

**Figure 2:**
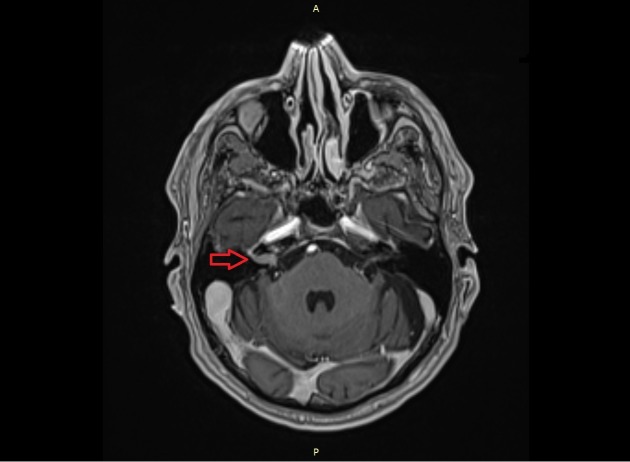
MRI scan. The image shows the rapid progress of the suspected vestibular schwannoma inside the right internal acoustic meatus. MRI: magnetic resonance imaging

**Figure 3:**
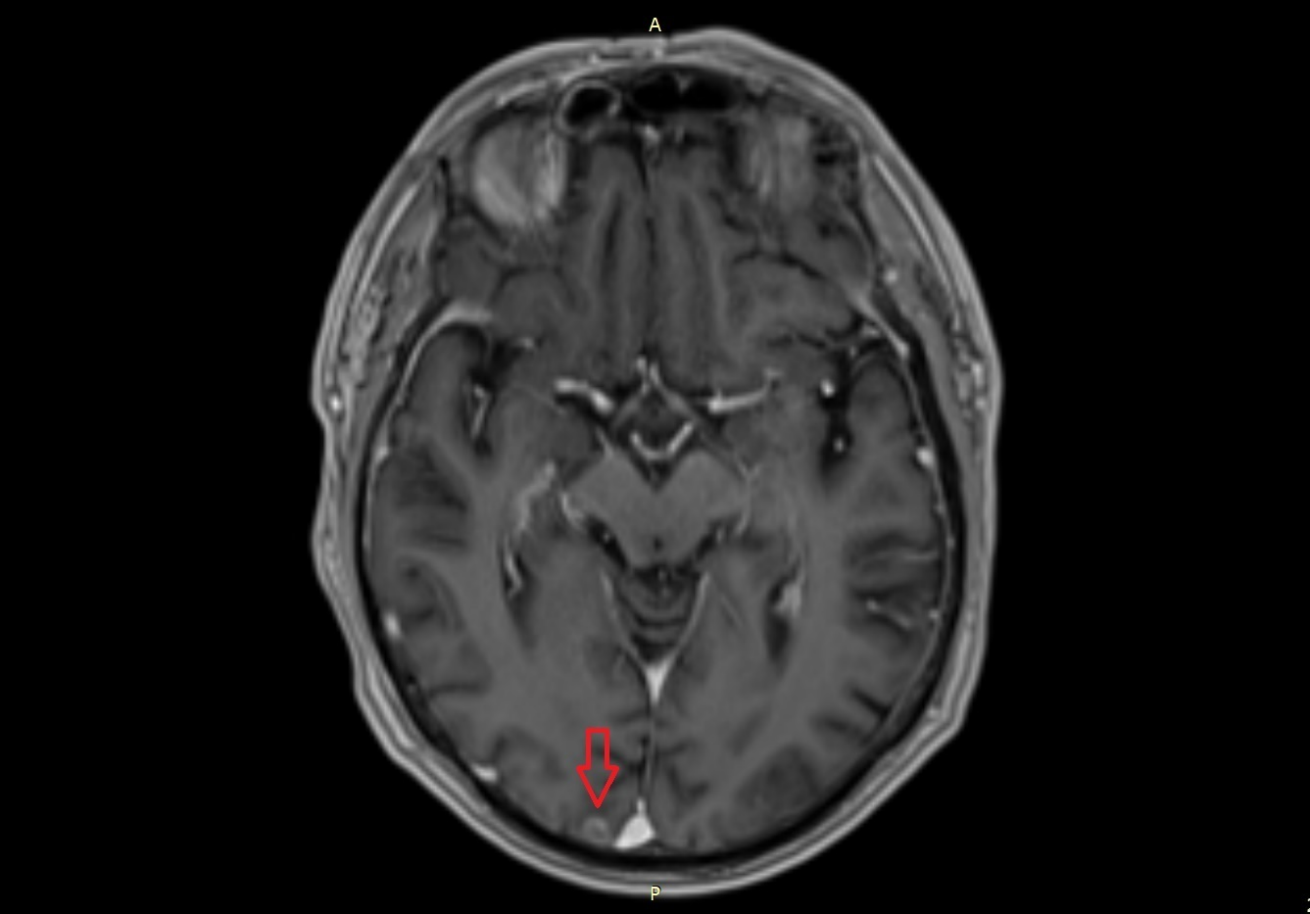
MRI scan. The image shows the suspect lesion in the right occipital lobe. MRI: magnetic resonance imaging

**Figure 4:**
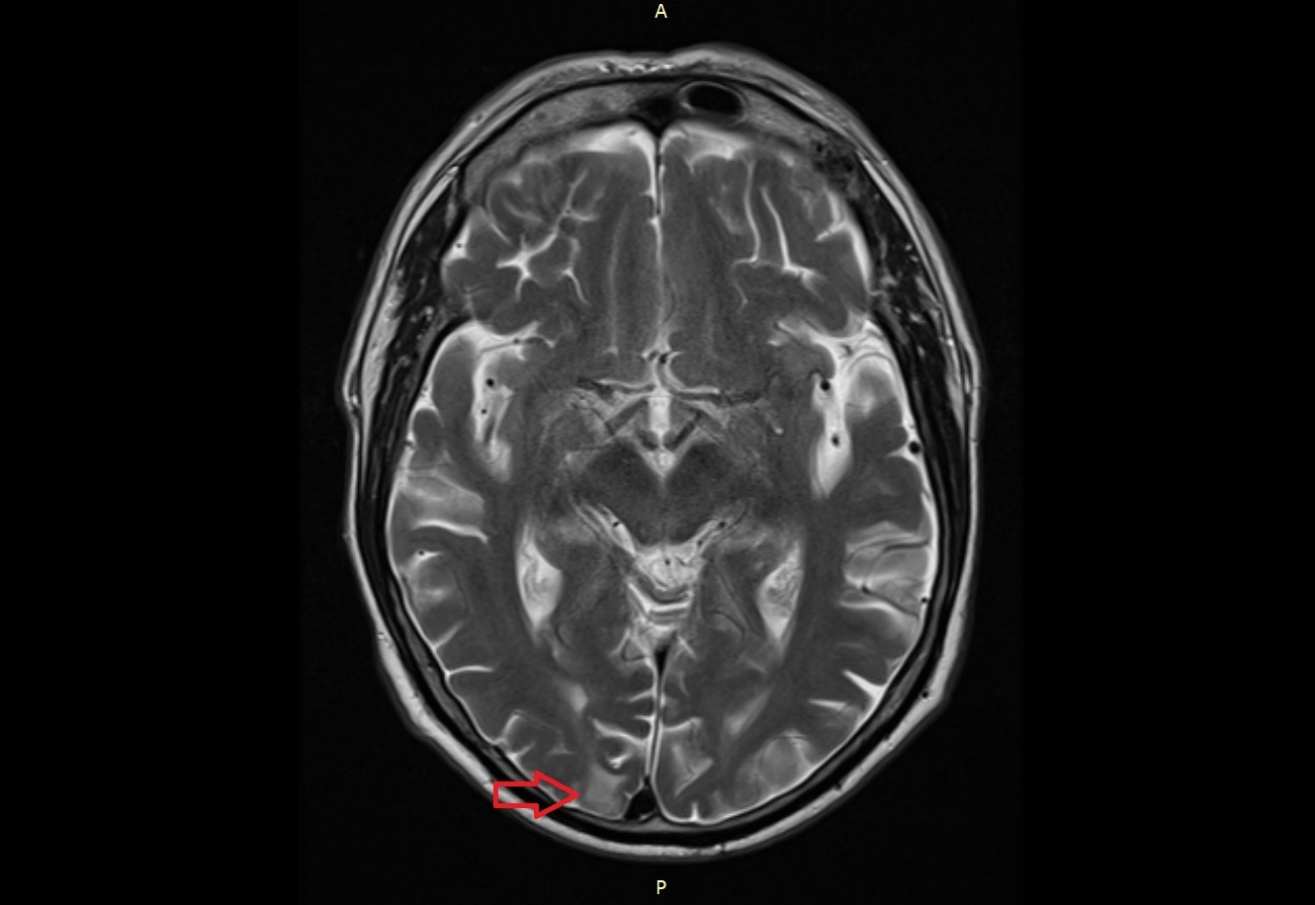
MRI scan. The image shows the suspect lesion in the right occipital lobe. MRI: magnetic resonance imaging

The new MRI scan showed a rapid growth of the suspected vestibular schwannoma from 5 x 5 x 4 mm two months ago to 12 x 5 x 4 mm (Figure 2). This quick progress is very atypical for a vestibular schwannoma [6]. Additionally, the radiologist found a suspect lesion in the right occipital lobe, directly located next to the superior sagittal sinus (Figure 3-4). The lesion was atypical for an ischemic infarct due to the specific contrast enhancement and morphology and was suspected to be a metastasis of an unknown primary tumor.

In agreement with the radiologist and radiosurgeon at the CyberKnife Center, the patient underwent a computed tomography (CT) scan of the neck, chest, and abdomen in search of the primary tumor. Furthermore, a biopsy of the occipital lesion and a lumbar puncture were performed at the Klinikum Großhadern Munich for histopathological analysis.

**Figure 5:**
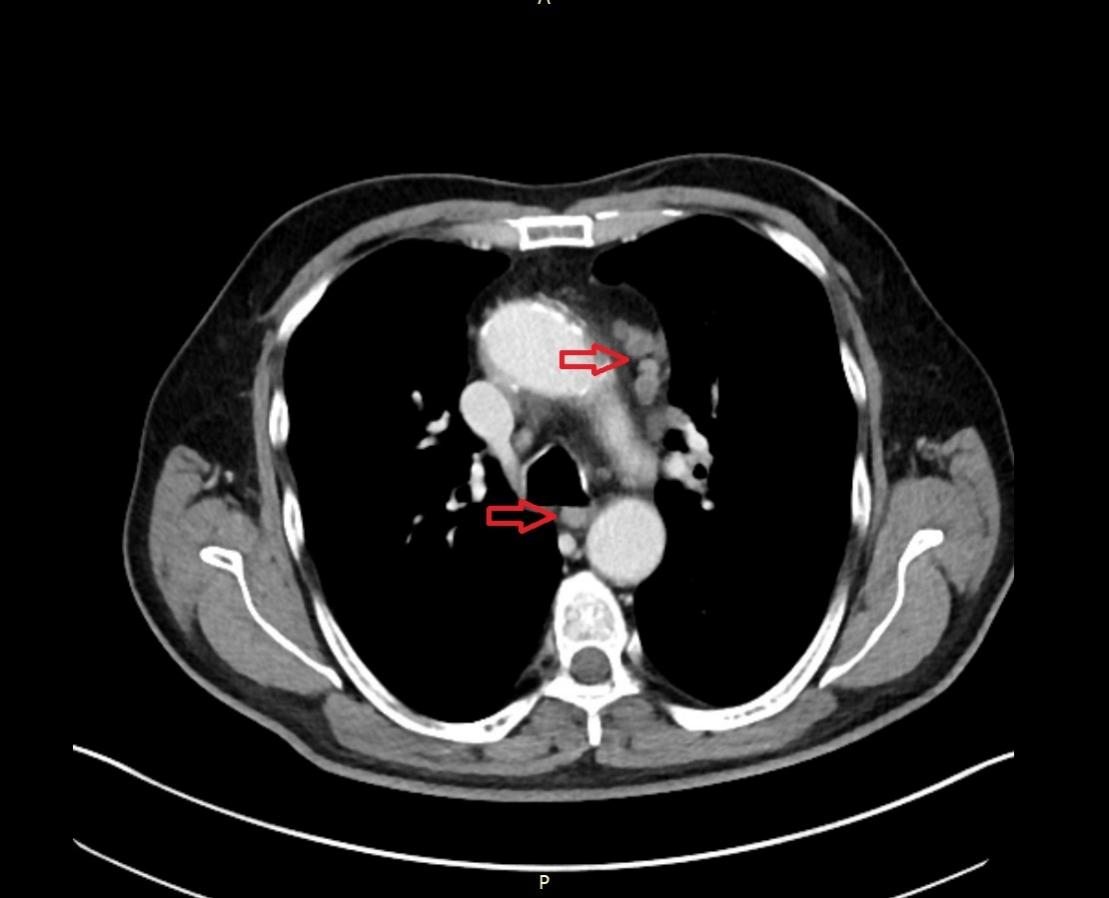
CT scan. The image shows suspect mediastinal lymph nodes. CT: computed tomography

**Figure 6:**
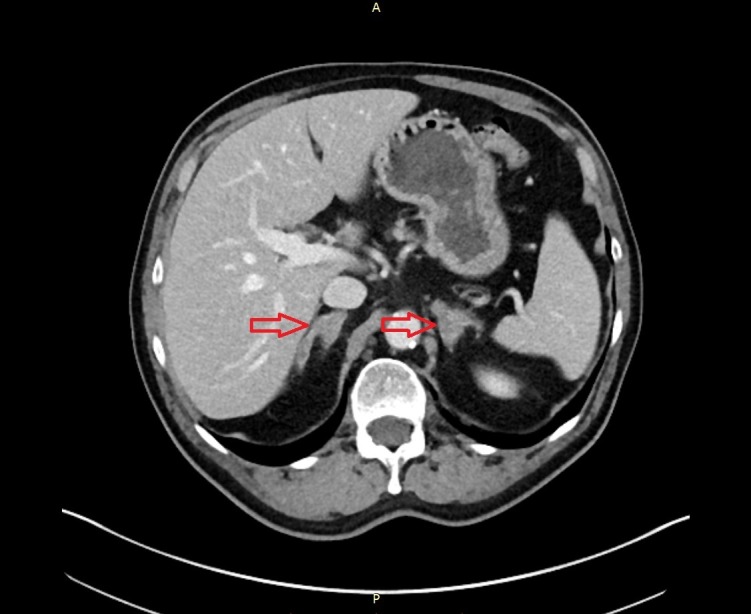
CT scan. The image shows both adrenal glands distended, probably caused by metastases. CT: computed tomography

**Figure 7:**
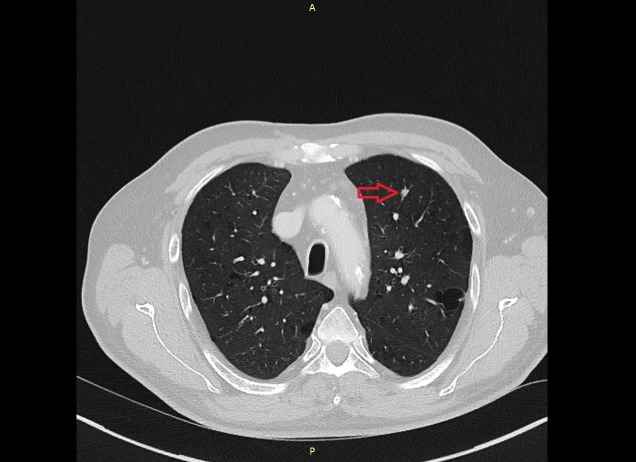
CT scan. The image shows the 6 x 6 mm lesion in the left upper lobe. CT: computed tomography

**Figure 8:**
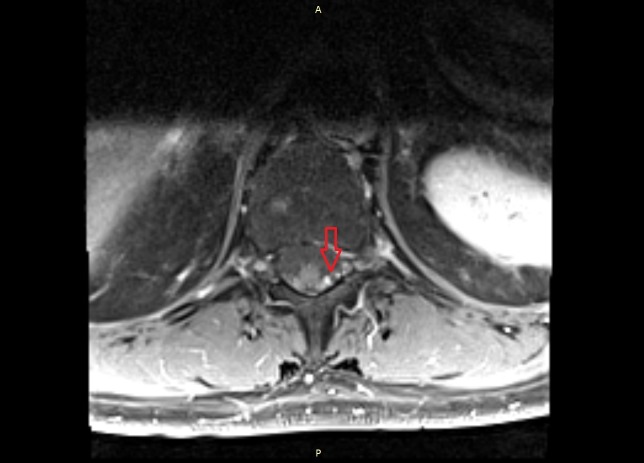
MRI scan. The image shows a probable meningeal carcinomatosis near the thoracolumbar transition. Dorsolateral of the myelon, a contrast-enhancing structure is visible. MRI: magnetic resonance imaging

The result of the lumbar puncture and the biopsy showed no malignant or suspect cells. However, the CT scan of the chest showed multiple suspicious supraclavicular and mediastinal lymph nodes (Figure 5). Moreover, the CT of the abdomen revealed questionable malignant lesions in both adrenal glands (Figure 6). In addition, a 6 x 6 mm lesion in the left upper lobe of the lung was detected (Figure 7). To complete the radiological assessment, a further MRI scan of the spine was performed. The scan showed a meningeal carcinomatosis in the cervical spine, near the thoracolumbar transition (Figure 8).

To secure the diagnosis, an ultrasound-guided biopsy of the mediastinal lymph nodes and another lumbar puncture were performed. Once again, the puncture exposed no suspicious cells. However, a histopathological examination of the lymph nodes showed small cell groups of a high-grade (G3) non-small cell lung cancer (NSCLC). Due to the low amount of malignant cells from the taken biopsy, a detailed molecular examination was not feasible.

After completing the diagnostics, the findings were discussed in the interdisciplinary tumor board of the Klinikum Großhadern Munich. The TNM stage classification of the NSCLC was – at least – cT1 cN3 cM1. Due to the advanced stage of the disease, a palliative treatment regimen was chosen. The patient agreed with the further proceedings, consisting of a whole-brain radiation with 5 x 3.0 Gy per week, 30 Gy in total, and an additional systemic chemotherapy.

## Discussion

A small vestibular schwannoma usually causes hearing loss, tinnitus, vertigo, and headaches [5]. A facial nerve palsy, especially a severe one, is a rare and uncommon symptom of small vestibular schwannomas [8]. In this case, we see an extremely uncommon dynamic of a tumor growth in the internal acoustic meatus. As mentioned, schwannomas, which are shown to grow, have an average growth of three mm per year [6]. The highest growth rate reported is up to 25 mm per year [9]. Here, the estimated growth rate of the tumor is about 42 mm per year, which suggests that the detected tumor is malignant.

However, the tumor has the radiological appearance of a schwannoma in terms of contrast-enhancement and localization. Furthermore, most of the symptoms caused can be well explained by a vestibular schwannoma. Additionally, a single NSCLC metastasis in the internal acoustic meatus is a highly unusual finding. To our knowledge, this is one of the very few reports of this kind of metastasis in the literature [10].

In one way or another, this case describes a very rare finding in terms of growth and dynamics in the internal acoustic meatus. It will be of high interest to see how the tumor will develop and react to radiation and chemotherapy. When this case report was written, the patient had not yet started his radiation and chemotherapy.

## Conclusions

This case emphasizes the importance of maintaining a high degree of awareness when tumors of the acoustic meatus are discovered. Despite the frequency and, often typical, radiological appearance, it is of utmost importance to carefully evaluate the patients’ symptom constellation, symptom severity, and their dynamic. Moreover, when a suspicious clinical appearance occurs, it is crucial to set up an interdisciplinary approach to determine further diagnostic and therapeutic approaches. Only in doing so will the patient receive the most suitable treatment for his condition.
